# The Impact of Methylenetetrahydrofolate Reductase C677T Polymorphism on Patients Undergoing Allogeneic Hematopoietic Stem Cell Transplantation with Methotrexate Prophylaxis

**DOI:** 10.1371/journal.pone.0163998

**Published:** 2016-10-26

**Authors:** Ja Min Byun, Hea-Lim Kim, Dong-Yeop Shin, Youngil Koh, Sung-Soo Yoon, Moon-Woo Seong, Sung Sup Park, Jin Hee Kim, Yun-Gyoo Lee, Inho Kim

**Affiliations:** 1 Department of Internal Medicine, Seoul National University College of Medicine, Seoul National University Hospital, Seoul, Korea; 2 Graduate School of Public Health, Seoul National University, Seoul, Korea; 3 Cancer Research Institute, Seoul National University, Seoul, Korea; 4 Department of Laboratory Medicine, Seoul National University Hospital, Seoul, Korea; 5 Department of Integrative Bioscience and Biotechnology, Sejong University, Seoul, Korea; 6 Department of Internal Medicine, Kangbuk Samsung Hospital, Sungkyunkwan University School of Medicine, Seoul, Korea; National Cancer Center, JAPAN

## Abstract

Pharmacogenomics can explain the inter-individual differences in response to drugs, including methotrexate (MTX) used for acute graft-versus-host disease (aGVHD) prophylaxis during hematopoietic stem cell transplantation (HSCT). In real-world practice, preplanned MTX dose is arbitrarily modified according to observed toxicity which can lead to unexpected and severe aGVHD development. We aimed to validate the influence of *MTHFR* C677T polymorphism on the outcomes of allogenic HSCT in a relatively under-represented homogenous Asian population. A total of 177 patients were divided into 677TT group versus 677C-carriers (677CT+677CC), and clinical outcomes along with baseline characteristics were analyzed and compared. Although there was a tendency towards increased peak liver function test results and accordingly greater delta values between the highest and the baseline in 677TT group, we found no associations between genotypes and hepatotoxicity. However, the incidence of acute liver GVHD (≥ grade 2) was significantly higher in the 677TT group than in the 677CC + 677CT group (*P* = 0.016). A total of 25 patients (14.1%) expired due to transplantation related mortality (TRM) during the first 180 days after HSCT. Patients carrying 677TT genotype were more likely to experience early TRM than 677C-carriers. The same pattern was observed in the cumulative TRM rate, and 677TT genotype patients were more prone to cumulative TRM (*P* = 0.010). This translated into shorter OS for patients with 677TT compared to 677C-carriers (*P* = 0.010). The 3-year survival after HSCT was 29.9% for 677TT cases and 47.1% for 677C-carriers. The multivariate analysis identified 677TT genotype (HR = 1.775. 95% CI 1.122–2.808, *P* = 0.014) and non-CR state (HR = 2.841. 95% CI 1.627–4.960, *P*<0.001) as predictors for survival. In conclusion, the *MTHFR* 677TT genotype appears to be associated with acute liver GVHD, and represent a risk factor for TRM and survival in patients undergoing HSCT with MTX as GVHD prophylaxis.

## Introduction

Methotrexate (MTX) is commonly used to prevent acute graft-versus-host disease (GVHD) after hematopoietic stem cell transplantation (HSCT) [1]. Given the inter-individual differences in the MTX metabolism, in real-world practice preplanned MTX dose is frequently modified according to observed toxicity. Unfortunately, such arbitrary schedule adaption of MTX dosage can lead to increased risk of acute GVHD that is both unexpected and severe [2].

The mechanism of action of MTX mainly consists of competitive inhibition of dihydrofolate reductase enzyme, thereby depleting reduced forms of tetrahydrofolate and contributing to cell death [3]. Moreover, the metabolites of MTX inhibit other folate enzymes, including 5,10-methylenetetrahydrofolate reductase (MTHFR), which is critical in apportioning folate substrates to downstream nucleotide synthesis and DNA methylation [4, 5]. The variation in MTX toxicity profiles are often attributed to inherited polymorphisms in genes encoding for drug-metabolizing enzymes, namely *MTHFR* 677C>T that reduces MTHFR activity and increases its thermolability [6]. It is also known that the prevalence of *MTHFR* C677T genotypes varies among different ethnic groups [7]. Previous studies, including our own, have investigated the relationship between polymorphisms affecting folate metabolism and MTX toxicity and/or efficacy [8–12] but the results were either non-confirmatory or non-reproducible. To this end, we aimed to validate the influence of *MTHFR* C677T polymorphism on the outcomes of allogenic HSCT in a relatively under-represented homogenous Asian population.

## Materials and Methods

### Study design and subjects

This was a retrospective longitudinal cohort study carried out at a single tertiary hospital. The data on adult patients receiving allogeneic HSCT at Seoul National University Hospital between January 2007 and December 2015 were collected. The patient eligibility criteria included (1) a first transplant; (2) age ≥ 18 years old; (3) three or four intravenous doses of MTX (three doses: days 1, 3, and 6 at 15 mg/m^2^; four doses: days 1 at 15 mg/m2 and days 3, 6, and 11 at 10 mg/m^2^) and cyclosporine (continuous infusion, starting on the day before transplantation) for GVHD prophylaxis; and (4) presence of informed consent to *MTHFR* genotyping. Exclusion criteria included (1) a second or subsequent transplant, (2) the cord blood HSCT, (3) GVHD prophylaxis without MTX, and (4) impaired baseline renal (creatinine clearance < 60 mL/min/1.73 m^2^) or hepatic (serum bilirubin > 3 mg/dL) function. The *MTHFR* C677T genotype was determined using polymerase chain reaction-restriction fragment length polymorphism analysis, as previously described [10]. All follow-up data available up until May 2016 were used. This study was conducted according to the Declaration of Helsinki and was approved by the institutional review board at Seoul National University (IRB No. H-1510-029-708).

### End point definitions

Based on previous reports including our own, the patients were divided into 677C-carriers (677CC+677CT) versus 677TT cases for comparison [10, 13]. Liver toxicity was defined by serum measurements total bilirubin, aspartic transaminase (AST), alanine transaminase (ALT), and alkaline phosphatase (ALP). The baseline liver function levels, peak level and differences between highest and baseline were taken into consideration. Veno-occlusive disease (VOD) was diagnosed based on development of hepatomegaly, weight gain and jaundice as described elsewhere [14]. Clinical outcomes measured included veno-occlusive disease (VOD), acute GVHD, transplantation-related mortality (TRM), chronic GVHD, relapse free survival (RFS) and overall survival (OS). Acute GVHD (aGVHD) was graded from 0 to 4 according to published data [15] from day 1 after HSCT, while chronic GVHD (cGVHD) was evaluated in patients who survived with sustained engraftment from day 100 after HSCT [16]. TRM was calculated from the time of transplantation to death related to transplant without relapse. Early TRM was calculated at day 180 after HSCT. OS was defined as the time from the date of HSCT to death of any cause while RFS was derived from the date of HSCT to that of relapse or death from any cause.

### Statistical analysis

The primary outcome of interest of this study was the development of MTX related toxicity in patients with different polymorphism status. By univariate analysis, odds ratios (OR) and 95% confidence intervals (95% CI) were used to estimate the risk of developing toxicity. By multivariate logistic regression analysis, adjusted OR were calculated, with the dependent variable being specific toxicity per involved site. The multivariate model included sex, age, primary diagnosis, conditioning regimen, donor status and the MTHFR polymorphisms as covariates and they were checked for possible interaction or confounding effects. If a covariate had an effect of 10% or more, then it was considered a confounding factor and the model was adjusted for it.

Univariate and multivariate proportional hazards regression models were used to identify independent risk factors of overall survival and treatment-related mortality by means of log-rank tests and Cox proportional hazards models, respectively. The survival curves were estimated using the Kaplan-Meier method. A stepwise backward procedure was used to construct a set of independent predictors of each end points. All predictors achieving a *P* value below 0.10 were considered, and sequentially removed if the *P* value in the multiple model was above 0.05. Differences between groups were assessed using a Student’s t-test or one-way analysis of variance for continuous variables, and Pearson chi-square test for categorical variables, as indicated. All data were analyzed using the Statistical Package for the Social Sciences software (IBM^®^ SPSS^®^ Statistics, version 22.0). *P* values of < 0.05 were considered statistically significant.

## Results

### Patient characteristics

The baseline characteristics of 177 Korean patients enrolled are described in Table 1. The frequency of *MTHFR* genotypes in decreasing order is as follows: 46.3% (82/177) for 677CT, 32.2% (57/177) for 677CC, and 21.5% (38/177) for 677TT. The mean age at HSCT was 37.8±12.5 years old, and there were more males (108, 61%) in the total cohort. Acute myeloid leukemia was the most common etiology (87, 49.2%) and acute leukemias including blast crisis of chronic myeloid leukemia constituted 90.4% (160/177) of the cohort.

**Table 1:** Baseline characteristics.

	Total (%)	MTHFR C677T polymorphism (%)		
		CC	CT	TT
**N**	177	57 (32.2)	82 (46.3)	38 (21.5)
**Age (years, mean**±**SD)**	37.8 (12.5)	38.8 (11.8)	36.9 (12.8)	38.2 (13.2)
**Sex (male, n)**	108 (61.0)	36 (63.2)	52 (63.4)	20 (52.6)
**Diagnosis**				
AML	87 (49.2)	31 (54.4)	38 (46.3)	18 (47.4)
ALL	61 (34.5)	17 (29.8)	28 (34.1)	16 (42.1)
Other acute leukemia	6 (3.4)	2 (3.5)	4 (4.9)	0
AA	3 (1.7)	1 (1.8)	1 (1.2)	1 (2.6)
MDS	8 (4.5)	4 (7.1)	3 (3.7)	1 (2.6)
Lymphoma	5 (2.8)	0	4 (4.9)	1 (2.6)
CML BC	6 (3.4)	2 (3.5)	3 (3.7)	1 (2.6)
MPN	1 (0.6)	0	1 (1.2)	0
**Conditioning regimen**				
Myeloablative	123 (69.5)	40 (70.2)	59 (72.0)	24 (63.2)
Non-myeloablative	54 (30.5)	17 (29.8)	23 (28.0)	14 (36.8)
**Stem cell source**				
Peripheral blood	171 (96.6)	54 (94.7)	82 (100)	35 (92.1)
Bone marrow	6 (3.4)	3 (5.3)	0	3 (7.9)
**Donor**				
Matched related	94 (53.1)	28 (49.1)	47 (57.3)	19 (50)
Mismatched related	13 (7.3)	3 (5.3)	3 (3.7)	7 (18.4)
Matched unrelated	39 (22.0)	12 (21.1)	23 (28.0)	4 (10.5)
Mismatched unrelated	31 (17.5)	14 (24.6)	9 (11.0)	8 (21.1)
**Disease status at HSCT**				
CR1	98 (55.4)	25 (43.9)	50 (61.0)	23 (60.5)
CR2	39 (22.0)	18 (31.6)	13 (15.9)	8 (21.1)
Beyond CR3	1 (0.6)	0	1 (1.2)	0
No treatment	1 (0.6)	0	1 (1.2)	0
Non-remission	38 (21.5)	14 (24.6)	17 (20.7)	7 (18.4)

SD, standard deviation; AML, acute myeloid leukemia; ALL, acute lymphoblastic leukemia; AA, aplastic anemia; MDS, myelodysplastic syndrome; CML BC, chronic myeloid leukemia blast crisis; MPN, myeloproliferative disease; HSCT, hematopoietic stem cell transplantation; CR1, first remission; CR2, second remission; CR3, third remission

### Liver toxicity and graft-versus-host disease

The results of serum liver function test results are presented in Table 2. There were no associations between differences in the baseline liver function between the groups. Although the peak total bilirubin, ALP and AST were the highest in 677TT group, the differences did not show statistical significance. VOD was diagnosed in 10 patients (5.6%). There were no patients who developed VOD in 677TT group, but the differences according to genotypes were not observed (*P* = 0.234).

**Table 2:** Hematopoietic stem cell transplantation (HSCT) outcomes.

	MTHFR C677T polymorphism (%)			*P*-value^1^
	CC	CT	TT	
**N**	57 (32.2)	82 (46.3)	38 (21.5)	NA
**VOD**	4 (7.0)	6 (7.3)	0	0.089
** Acute GVHD**				
Any	12 (21.1)	23 (28.0)	11 (28.9)	0.639
Skin (≥grade 2)	11 (19.3)	17 (20.7)	5 (13.2)	0.327
Liver(≥grade 2)	2 (3.5)	7 (8.5)	8 (21.1)	0.007
Gastrointestinal(≥grade 2)	4 (7.0)	9 (11.0)	6 (15.8)	0.256
** Chronic GVHD**	22 (38.6)	35 (42.7)	11 (28.9)	0.133
** Outcomes of HSCT**				
No relapse	17 (29.8)	42 (51.2)	12 (31.6)	0.226
Early TRM	8 (14.0)	7 (8.5)	10 (26.3)	0.015
Cumulative TRM	11 (19.3)	14 (17.1)	39 (22.0)	0.013
**Baseline LFT**				
Total bilirubin (mg/dL)	0.7 (0.5)	0.7 (0.3)	0.8 (0.4)	0.411
ALP (IU/L)	65.5 (22.0)	68.7 (29.5)	70.3 (29.4)	0.556
AST (IU/L)	42.8 (76.0)	40.8 (49.0)	34.2 (20.5)	0.466
ALT (IU/L)	56.5 (83.4)	54.8 (74.2)	52.6 (51.6)	0.832
**Peak LFT**				
Total bilirubin (mg/dL)	3.6 (6.5)	3.1 (5.0)	4.6 (7.1)	0.245
ALP (IU/L)	147.2 (102.6)	141.4 (92.6)	209.9 (291.4)	0.176
AST (IU/L)	139.2 (225.0)	124.4 (201.2)	239.8 (589.0)	0.268
ALT (IU/L)	195.4 (265.3)	154.2 (135.1)	177.2 (231.2)	0.874
**Differences in LFT^2^**				
ΔTotal bilirubin (mg/dL)	2.9 (6.4)	2.4 (4.9)	3.8 (7.0)	0.262
ΔALP (IU/L)	81.7 (98.2)	74.0 (82.3)	140.5 (291.9)	0.195
ΔAST (IU/L)	103.6 (226.7)	89.5 (201.8)	207.6 (587.9)	0.254
ΔALT (IU/L)	141.5 (252.5)	106.4 (132.2)	130.9 (233.0)	0.784

NA, not applicable; VOD, veno-occlusive disease; GVHD, graft-versus-host disease; TRM, transplantation related mortality; LFT, liver function test; ALP, alkaline phosphatase; AST, asparate aminotransferase; ALT, alanine aminotransferase

^1^*P*-value was calculated for 677CC+677CT versus 677TT

^2^Differences between the baseline value and the peak value for each patient

Acute GVHD of any grades occurred in 46 patients (26.0%), and there were no differences in the overall incidence of aGVHD between different genotypes. However, the incidence of acute liver GVHD (≥ grade 2) was significantly higher in the 677TT group than in the 677CC+677CT group (*P* = 0.016). Multivariate analysis (Table A in S1 Appendix) confirmed that 677TT is more often associated with acute liver GVHD compared to 677CC + 677CT group (OR = 3.480, 95% CI 1.218–9.941, *P* = 0.020). There were no differences in the cumulative incidence of cGVHD between the two groups.

### Transplantation related mortality

A total of 25 patients (14.1%) expired due to TRM during the first 180 days after HSCT. The main cause of TRM was infection (19, 76.0%), followed by graft failure in 5 patients (20.0%) and hemorrhage in 1 patient (4.0%). Patients carrying 677TT genotype were more likely to experience early TRM than 677C-carriers (*P* = 0.019, Fig A in S2 Appendix). There were no association between infection, the major cause of TRM, and different polymorphism status (*P* = 0.572, data not shown).

**Fig 1:**
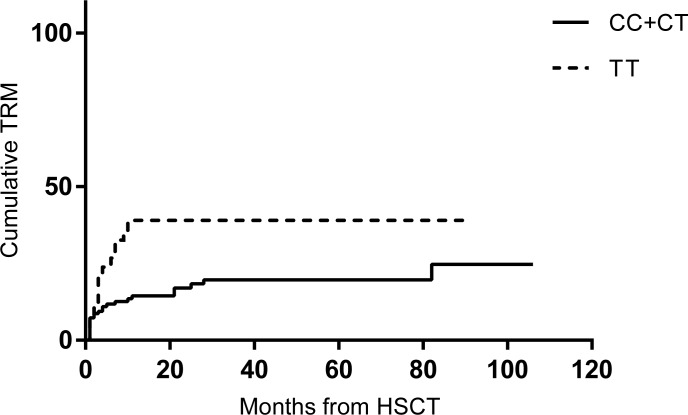
Cumulative transplantation related morality according to MTHFR C677T genotype (*P* = 0.010).

During the median follow-up of 30 months, there were 64 (36.2%) cumulative cases of TRM. The causes of TRM are as follows: infection in 50 patients (78.1%), GVHD in 7 (10.9%), graft failure 5 (7.8%), and hemorrhage in 2 (3.1%). The MTHFR C677T polymorphism status was not associated with infection in this setting either (*P* = 0.949, data not shown). 677TT genotype patients were more prone to cumulative TRM (*P* = 0.010, Fig 1). Through multivariate analyses (Table 3), 677TT genotype (HR = 2.539, 95% CI 1.300–4.958, *P* = 0.006) and pre-HSCT disease status (HR = 2.910. 95% CI 1.247–6.792, *P* = 0.013) were identified as risk factors for cumulative TRM.

**Table 3:** Multivariate analyses for predictors of overall survival and treatment related mortality.

Outcomes	Variables increasing the risk of outcomes	Hazards ratio (95% CI)	*P*-value	
**Cumulative TRM**	MTHFR genotype (CC+CT vs TT)	2.539 (1.300–4.958)		0.006
	Disease status at HSCT (CR vs non-CR)	2.910 (1.247–6.792)		0.013
**Overall survival**	MTHFR genotype (CC+CT vs TT)	1.775 (1.122–2.808)		0.014
	Disease status at HSCT (CR vs non-CR)	2.841 (1.627–4.960)		<0.001

CI, confidence intervals; HSCT, hematopoietic stem cell transplantation; CR, complete remission; TRM, transplantation related mortality

### Relapse and overall survival

**Fig 2:**
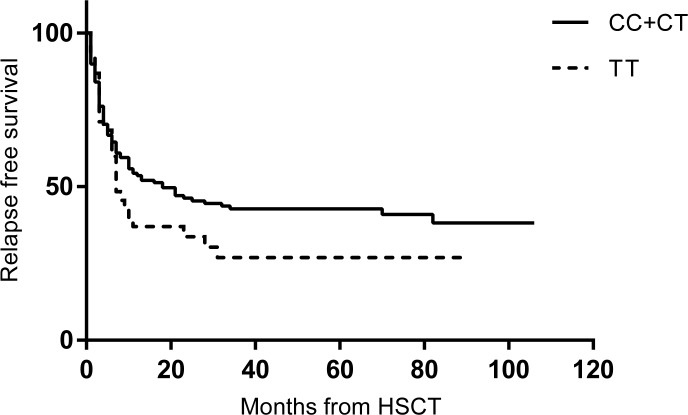
Relapse free survival according to MTHFR C677T genotype (*P* = 0.168).

During the median follow-up period of 30 months (range 3–141), 71 patients (40.1%) remained relapse free after HSCT and 78 patients (44.1%) remained alive. The median RFS was 20 months and median OS 21 months for the whole population. With regard to the impact of the C677T polymorphism on RFS, RFS did not differ significantly between patients with 677TT versus 677CT + 677CC genotypes (Fig 2, *P* = 0.168).

**Fig 3:**
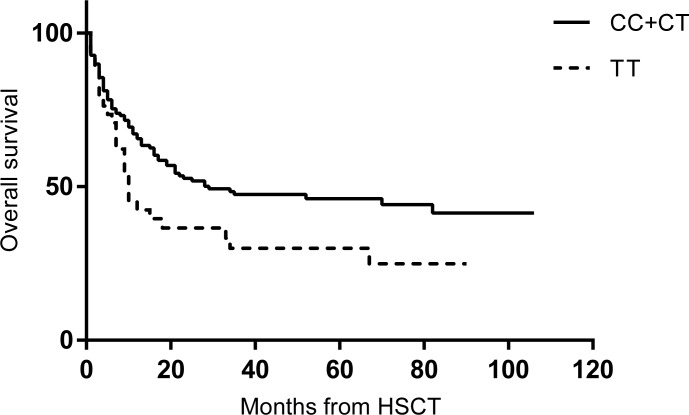
Overall survival according to MTHFR C677T genotype (*P* = 0.036).

On the other hand, patients with 677TT showed shorter OS compared to 677C-carriers (Fig 3, *P* = 0.010). The 3-year survival after HSCT was 29.9% for 677TT cases and 47.1% for 677C-carriers. The multivariate analysis identified 677TT genotype (HR = 1.775. 95% CI 1.122–2.808, *P* = 0.014) and non-CR state (HR = 2.841. 95% CI 1.627–4.960, *P*<0.001) as predictors for survival (Table 3).

## Discussion

Allogeneic hematopoietic stem cell transplantation offers chance of cure in many hematologic diseases. However, the donor effect or immune responses to allogeneic and autologous antigens can cause GHVD, and this remains the major toxicity of this otherwise effective therapeutic option [17]. Transplantation related mortality, especially early TRM occurring within 100–180 days of HSCT, also presents a clinical challenge for many hematologists. Gene polymorphisms interfering with metabolism of drugs used during HSCT have been studied as potential predictors for GVHD occurrence and survival. We selected *MTHFR* C677T, a particular gene with a high profile of being involved in methotrexate metabolism, and aimed to evaluate its impact on the outcomes of allogenic HSCT. This is one of the few attempts to investigate the role of *MTHFR* in a relatively under-represented homogenous Asian population. We found that patients harboring 677TT genotype developed acute liver GVHD more frequently compared to their 677C-ccarrying counterparts (*P* = 0.007). Patients with 677TT genotype were also at increased risk of TRM by approximately 2.5 folds (95% CI 1.300–4.958, *P* = 0.006), and associated with lower overall survival (*P* = 0.036).

The most common genotype in our cohort was 677CT (46.3%) and such genotype frequencies were similar to those previously reported in Asian population [18]. An interesting finding of the present study was that the *MTHFR* 677TT genotype was a risk factor for acute liver GVHD. Several studies have investigated the effect of *MTHFR* C677T on overall acute GVHD but have, however, shown inconsistent results [19–22]. While some studies have revealed a protective effect of *MTHFR* C677T on acute GVHD [19, 20], others have shown either opposing results [21], or were not able to detect an association between acute GVHD and the C677T polymorphisms [22]. We did not find any association between the incidence of overall acute GVHD and the *MTHFR* C677T polymorphism. However, when analyzing organ-specific acute GVHD, there was a significant association between the C677T genotype and acute liver GVHD. Considering that the development of acute GVHD can be affected by the toxicity of the preparative regimen [23] and that *MTHFR* is an essential enzyme in DNA synthesis and methylation, a delayed repair from cell damage caused by MTX in patients with the 677TT genotype can partly explain our findings. Also, given that all of our patients received MTX via intravenous, it seems plausible that liver suffered the highest intracellular concentration of MTX leading to additional damage compared to more peripheral organ structures like skin.

Another considerable result of our study is that TRM and OS of patients with the 677TT genotype were significantly inferior to 677C-carriers. 677TT genotype was also recognized as predictor for both TRM and OS through multivariate analysis. The patients undergoing HSCT are generally at high risk of folate deficiency due to decreased dietary intake and increased folate requirements. On top of this, patients with 677TT genotypes show more disturbed folate pathway compared to 677C-carriers [5, 24]. Thus these patients experience greater disruption of DNA methylation, changes in transcriptional regulation, increase in homocysteine levels, and ultimately altered nucleotide pools [24]. Such reduced DNA repair capacity in the 677TT genotype might result in delayed healing after HSCT, resulting in inferior TRM and OS.

Our results depicting no association between the *MTHFR* C677T polymorphism and MTX hepatotoxicity contradicts our previous findings. More specifically, although there was a tendency towards increased peak values and accordingly greater delta values (between the highest and the baseline), the difference did not reach statistical significance. Current findings are, however, in concordance with previous studies. Namely, Kalayoglu-Besisik et al. showed that patients with the *MTHFR* 677TT genotype have a tendency to experience higher MTX toxicity, but their data showed no statistically significant difference [9]. Also, Ulrich et al. failed to show an association between the *MTHFR* C677T genotype and total bilirubin levels after HSCT [12]. We cannot, at this point, decide our stance regarding the effects of *MTHFR* C677T polymorphisms on hepatotoxicity following MTX use in HSCT setting. Repeated investigations with larger number of patients are needed to confirm the association.

One of the major pitfalls of our study is the lack of donor polymorphism status. Also, status of other candidate gene polymorphisms including *MTHFR* A1298C were not evaluated. However, such lack of information does not diminish the clinical significance of our current study. Subsequent more intricately structured study, ideally prospective in nature, should follow to corroborate and explore our findings.

In conclusion, the *MTHFR* 677TT genotype appears to be associated with acute liver GVHD, and represent a risk factor for TRM and survival in patients undergoing HSCT with MTX as GVHD prophylaxis. Our findings from a homogenous Asian population can contribute to accumulating understanding of inter-individual variability in drug toxicity and efficacy, and ultimately to optimizing individualized approaches to GVHD prophylaxis for improved HSCT final outcomes.

## Supporting Information

S1 Appendix Table A: Toxicities associated with MTHFR C677T polymorphisms.
(PDF)

S2 Appendix F ig A: Early transplantation related mortality according to MTHFR C677T genotype (*P* = 0.019).
(PDF)

## References

[1] Storb R, Deeg HJ, Whitehead J, Appelbaum F, Beatty P, Bensinger W, et al Methotrexate and cyclosporine compared with cyclosporine alone for prophylaxis of acute graft versus host disease after marrow transplantation for leukemia. N Engl J Med. 1986;314(12):729–35. 10.1056/NEJM198603203141201 3513012

[2] Kumar S, Wolf RC, Chen MG, Gastineau DA, Gertz MA, Inwards DJ, et al Omission of day +11 methotrexate after allogeneic bone marrow transplantation is associated with increased risk of severe acute graft-versus-host disease. Bone marrow transplantation. 2002;30(3):161–5. 10.1038/sj.bmt.1703616 12189534

[3] Longo-Sorbello GS, Bertino JR. Current understanding of methotrexate pharmacology and efficacy in acute leukemias. Use of newer antifolates in clinical trials. Haematologica. 2001;86(2):121–7. 11224479

[4] Baggott JE, Vaughn WH, Hudson BB. Inhibition of 5-aminoimidazole-4-carboxamide ribotide transformylase, adenosine deaminase and 5'-adenylate deaminase by polyglutamates of methotrexate and oxidized folates and by 5-aminoimidazole-4-carboxamide riboside and ribotide. Biochem J. 1986;236(1):193–200. 2431676 10.1042/bj2360193 PMC1146805

[5] Friso S, Choi SW, Girelli D, Mason JB, Dolnikowski GG, Bagley PJ, et al A common mutation in the 5,10-methylenetetrahydrofolate reductase gene affects genomic DNA methylation through an interaction with folate status. Proc Natl Acad Sci USA. 2002;99(8):5606–11. 10.1073/pnas.062066299 11929966 PMC122817

[6] Frosst P, Blom HJ, Milos R, Goyette P, Sheppard CA, Matthews RG, et al A candidate genetic risk factor for vascular disease: a common mutation in methylenetetrahydrofolate reductase. Nat Genet. 1995;10(1):111–3. 10.1038/ng0595-111 7647779

[7] Almawi WY, Finan RR, Tamim H, Daccache JL, Irani-Hakime N. Differences in the frequency of the C677T mutation in the methylenetetrahydrofolate reductase (MTHFR) gene among the Lebanese population. Am J Hematol. 2004;76(1):85–7. 10.1002/ajh.20047 15114606

[8] Chiusolo P, Reddiconto G, Casorelli I, Laurenti L, Sora F, Mele L, et al Preponderance of methylenetetrahydrofolate reductase C677T homozygosity among leukemia patients intolerant to methotrexate. Ann Oncol. 2002;13(12):1915–8. 12453860 10.1093/annonc/mdf322

[9] Kalayoglu-Besisik S, Caliskan Y, Sargin D, Gurses N, Ozbek U. Methylenetetrahydrofolate reductase C677T polymorphism and toxicity in allogeneic hematopoietic cell transplantation. Transplantation. 2003;76(12):1775–7. 10.1097/01.TP.0000093831.63661.DF 14688536

[10] Kim I, Lee KH, Kim JH, Ra EK, Yoon SS, Hong YC, et al Polymorphisms of the methylenetetrahydrofolate reductase gene and clinical outcomes in HLA-matched sibling allogeneic hematopoietic stem cell transplantation. Ann Hematol. 2007;86(1):41–8. 10.1007/s00277-006-0184-3 17028897

[11] Soydan E, Topcuoglu P, Dalva K, Arat M. The impact of methylenetetrahydrofolate reductase (MTHFR) C677T gene polymorphism on transplant-related variables after allogeneic hematopoietic cell transplantation in patients receiving MTX as GVHD prophylaxis. Bone marrow transplantation. 2008;42(6):429–30. 10.1038/bmt.2008.184 18587432

[12] Ulrich CM, Yasui Y, Storb R, Schubert MM, Wagner JL, Bigler J, et al Pharmacogenetics of methotrexate: toxicity among marrow transplantation patients varies with the methylenetetrahydrofolate reductase C677T polymorphism. Blood. 2001;98(1):231–4. 11418485 10.1182/blood.v98.1.231

[13] Ongaro A, De Mattei M, Della Porta MG, Rigolin G, Ambrosio C, Di Raimondo F, et al Gene polymorphisms in folate metabolizing enzymes in adult acute lymphoblastic leukemia: effects on methotrexate-related toxicity and survival. Haematologica. 2009;94(10):1391–8. 10.3324/haematol.2009.008326 19648163 PMC2754955

[14] McDonald GB, Hinds MS, Fisher LD, Schoch HG, Wolford JL, Banaji M, et al Veno-occlusive disease of the liver and multiorgan failure after bone marrow transplantation: a cohort study of 355 patients. Ann Intern Med. 1993;118(4):255–67. 8420443 10.7326/0003-4819-118-4-199302150-00003

[15] Przepiorka D, Weisdorf D, Martin P, Klingemann HG, Beatty P, Hows J, et al 1994 Consensus Conference on Acute GVHD Grading. Bone marrow transplantation. 1995;15(6):825–8. 7581076

[16] Filipovich AH. Diagnosis and manifestations of chronic graft-versus-host disease. Best Pract Res Clin Haematol. 2008;21(2):251–7. 10.1016/j.beha.2008.02.008 18503990

[17] Koreth J, Ritz J. Tregs, HSCT, and acute GVHD: up close and personal. Blood. 2013;122(10):1690–1. 10.1182/blood-2013-07-514125 24009174 PMC3765054

[18] Urano W, Taniguchi A, Yamanaka H, Tanaka E, Nakajima H, Matsuda Y, et al Polymorphisms in the methylenetetrahydrofolate reductase gene were associated with both the efficacy and the toxicity of methotrexate used for the treatment of rheumatoid arthritis, as evidenced by single locus and haplotype analyses. Pharmacogenetics. 2002;12(3):183–90. 11927833 10.1097/00008571-200204000-00002

[19] Robien K, Bigler J, Yasui Y, Potter JD, Martin P, Storb R, et al Methylenetetrahydrofolate reductase and thymidylate synthase genotypes and risk of acute graft-versus-host disease following hematopoietic cell transplantation for chronic myelogenous leukemia. Biology of blood and marrow transplantation: journal of the American Society for Blood and Marrow Transplantation. 2006;12(9):973–80. 10.1016/j.bbmt.2006.05.016 16920564

[20] Sugimoto K, Murata M, Onizuka M, Inamoto Y, Terakura S, Kuwatsuka Y, et al Decreased risk of acute graft-versus-host disease following allogeneic hematopoietic stem cell transplantation in patients with the 5,10-methylenetetrahydrofolate reductase 677TT genotype. Int J Hematol. 2008;87(5):451–8. 10.1007/s12185-008-0061-z 18365141

[21] Rocha V, Porcher R, Fernandes JF, Filion A, Bittencourt H, Silva W Jr., et al Association of drug metabolism gene polymorphisms with toxicities, graft-versus-host disease and survival after HLA-identical sibling hematopoietic stem cell transplantation for patients with leukemia. Leukemia. 2009;23(3):545–56. 10.1038/leu.2008.323 19005482

[22] Pihusch M, Lohse P, Reitberger J, Hiller E, Andreesen R, Kolb HJ, et al Impact of thrombophilic gene mutations and graft-versus-host disease on thromboembolic complications after allogeneic hematopoietic stem-cell transplantation. Transplantation. 2004;78(6):911–8. 15385813 10.1097/01.tp.0000136988.38919.fb

[23] Couriel DR, Saliba RM, Giralt S, Khouri I, Andersson B, de Lima M, et al Acute and chronic graft-versus-host disease after ablative and nonmyeloablative conditioning for allogeneic hematopoietic transplantation. Biology of blood and marrow transplantation: journal of the American Society for Blood and Marrow Transplantation. 2004;10(3):178–85. 10.1016/j.bbmt.2003.10.006 14993883

[24] Blount BC, Ames BN. DNA damage in folate deficiency. Baillieres Clin Haematol. 1995;8(3):461–78. 8534957 10.1016/s0950-3536(05)80216-1

