# Supplementary material for: The Impact of Methylenetetrahydrofolate Reductase C677T Polymorphism on Patients Undergoing Allogeneic Hematopoietic Stem Cell Transplantation with Methotrexate Prophylaxis
Source: PLoS One. 2016 Oct 26;11(10):e0163998. doi: 10.1371/journal.pone.0163998 (PMC5081210; doi:10.1371/journal.pone.0163998)
Supplement: S1 Appendix — Table A: Toxicities associated with MTHFR C677T polymorphisms. (PDF) [file pone.0163998.s001.pdf]

(S1 Appendix) Table A. Toxicities associated with MTHFR C677T polymorphisms

	Univariate analysis[1]		Multivariate analysis[1]	
	OR (95% CI)	*P*-value	OR (95% CI)	*P*-value
**Acute GVHD**				
Any	1.759 (0.882-3.509)	0.109	1.311 (0.638-2.6919	0.461
Skin	0.996 (0.380-2.607)	0.993	0.755 (0.281-2.027)	0.577
Liver	4.615 (1.721-12.376)	0.002	3.480 (1.218-9.941)	0.020
Gastrointestinal	2.808 (1.030-7.654)	0.044	2.443 (0.838-7.121)	0.102
**Chronic GVHD**	1.228 (0.636-2.39)	0.540	1.102 (0.556-2.181)	0.781

OR, odds ratio; GVHD, graft-versus-host disease

[1]*P*-value was calculated for 677CC+677CT versus 677TT
