# Supplementary figures and images for: The Impact of Methylenetetrahydrofolate Reductase C677T Polymorphism on Patients Undergoing Allogeneic Hematopoietic Stem Cell Transplantation with Methotrexate Prophylaxis
Source: PLoS One. 2016 Oct 26;11(10):e0163998. doi: 10.1371/journal.pone.0163998 (PMC5081210; doi:10.1371/journal.pone.0163998)

(S2 Appendix) **Fig A.** Early transplantation related mortality according to MTHFR C677T genotype ( $P=0.019$ )

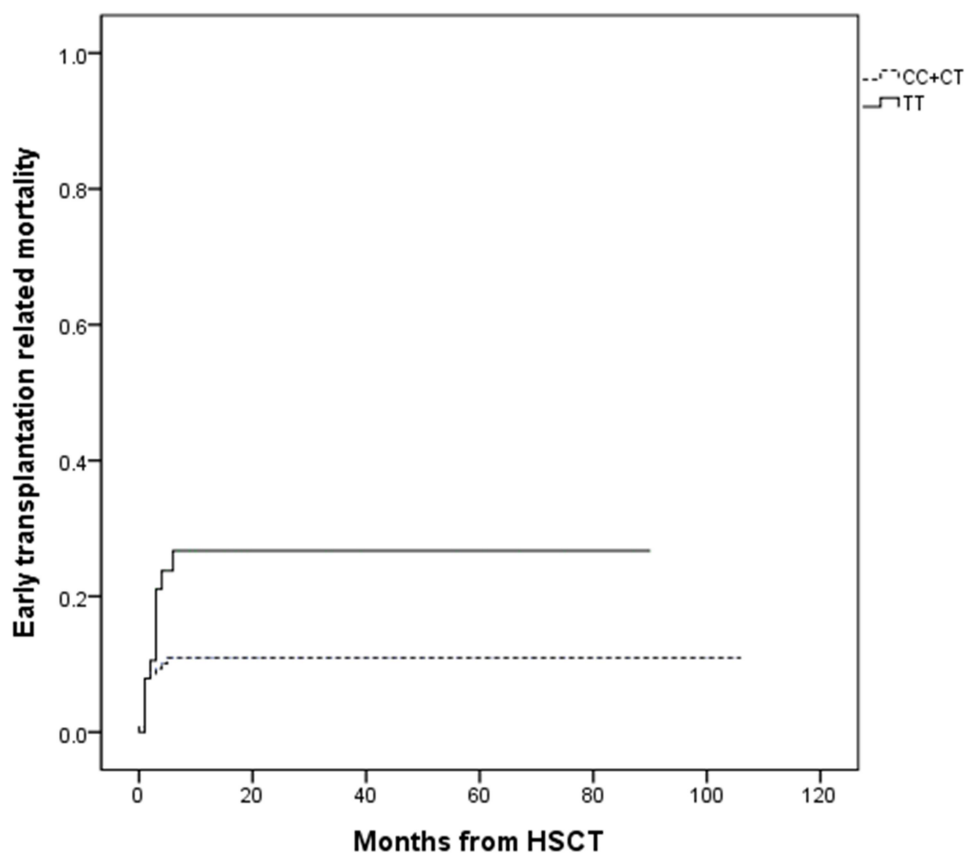

Supplement: S2 Appendix — ig A: Early transplantation related mortality according to MTHFR C677T genotype (P = 0.019). (PDF) [file pone.0163998.s002.pdf]
